# Mapping the future: bibliometric insights into ferroptosis and diabetic nephropathy

**DOI:** 10.3389/fphys.2025.1516466

**Published:** 2025-04-10

**Authors:** Tangwen Wei, Yang Qin, Xiaohui Lin, Xiujuan Wang, Suyi Chen, Xia Chen, Nan Yan, Xinyi Wei, Zhichang Zhang, Bing Wei

**Affiliations:** ^1^ Affiliated Hospital of Guilin Medical University, Guilin Medical University, Guilin, Guangxi, China; ^2^ Affiliated Hospital of Guilin Medical University, Guilin, Guangxi, China; ^3^ School of Laboratory Medicine, Guilin Medical University, Guilin, Guangxi, China; ^4^ Department of Academic Affairs, Guilin Medical University, Guilin, Guangxi, China; ^5^ Department of Medical Applied Technology, Shenyang Medical College, Shenyang, China; ^6^ School of Public Health, Guangxi Medical University, Nanning, Guangxi, China; ^7^ Department of Computer Science, College of Intelligent Medicine, China Medical University, Shenyang, China

**Keywords:** ferroptosis, diabetic nephropathy, bibliometric analysis, visual analysis, GPX4

## Abstract

**Background:**

Diabetic nephropathy (DN), a leading cause of end-stage renal disease, exerts a substantial burden on healthcare systems globally. Emerging evidence highlights ferroptosis - an iron-dependent form of cell death driven by lipid peroxidation and glutathione depletion - as a critical contributor to DN progression via oxidative stress, tubular injury, and glomerular dysfunction. Despite increasing research interest, a comprehensive synthesis of research trends and mechanistic insights is lacking.

**Objective:**

This study integrated bibliometric analysis with a mechanistic review to map the evolving ferroptosis landscape in DN, identify research hotspots, and propose future directions for therapeutic development.

**Methods:**

In total, 86 publications (2018–2023) were retrieved from the Web of Science Core Collection and analyzed using CiteSpace and VOSviewer. Co-occurrence networks, citation trends, and keyword bursts were examined to delineate global contributions, collaborative networks, and emerging themes.

**Results:**

Annual publication numbers surged 12-fold after 2020, with China contributing the highest proportion (60.4%), and led by institutions such as Zhengzhou University. The United States of America and Germany showed high centrality in collaborative networks. Key research themes included glutathione peroxidase 4 (GPX4)-mediated antioxidant defenses, acyl-CoA synthetase long-chain family member 4 (ACSL4)-mediated lipid remodeling, and iron dysregulation. *Frontiers in Endocrinology* (nine articles) and *Free Radical Biology and Medicine* (highest citation count: 171) emerged as pivotal publication platforms. Mechanistic analyses identified three ferroptosis defense axes (GPX4, FSP1/CoQ10, and GCH1/BH4) and cell type-specific vulnerabilities in tubular, podocyte, and endothelial cells. Preclinical agents, including ginkgolide B (GB) and dapagliflozin, effectively restored iron homeostasis and attenuated oxidative damage.

**Conclusion:**

Ferroptosis is a promising therapeutic target for DN, yet its clinical translation remains in its infancy. Future efforts should prioritize large-scale clinical trials, single-cell mechanistic profiling, and interdisciplinary integration to bridge molecular insights with precision therapies. This study provides a roadmap for advancing ferroptosis-targeted interventions for DN, emphasizing global collaborations and biomarker-driven strategies.

## 1 Introduction

Diabetic nephropathy is a major diabetic complication affecting 30%–50% of patients worldwide (Liu et al., 2020). The disease progresses from early manifestations, including glomerular hypertrophy and basement membrane thickening, to advanced stages characterized by mesangial proliferation and diffuse capillary lesions (Silva et al., 2020). The global diabetes epidemic, as reported by the International Diabetes Federation, has underscored the growing DN burden, with approximately 463 million adults aged 20–79 years diagnosed with diabetes in 2019. Emerging evidence now implicates ferroptosis - an iron-dependent cell death pathway characterized by reactive oxygen species (ROS) accumulation and lipid peroxidation (Zizzi et al., 2024; Stockwell, 2022) - as a key driver of DN pathogenesis. Although antioxidants and iron chelation therapies have demonstrated potential in mitigating oxidative stress in diabetic kidney disease, gaps remain in our understanding of the cell-type-specific ferroptosis triggers and clinically relevant regulatory targets (Gogebakan et al., 2012; Habib, 2018; Zhang et al., 2024). A systematic mechanistic investigation is therefore essential to bridge current pathophysiological insights with the development of precision-targeted interventions.

## 2 Materials and methods

### 2.1 Materials

**FIGURE 1:**
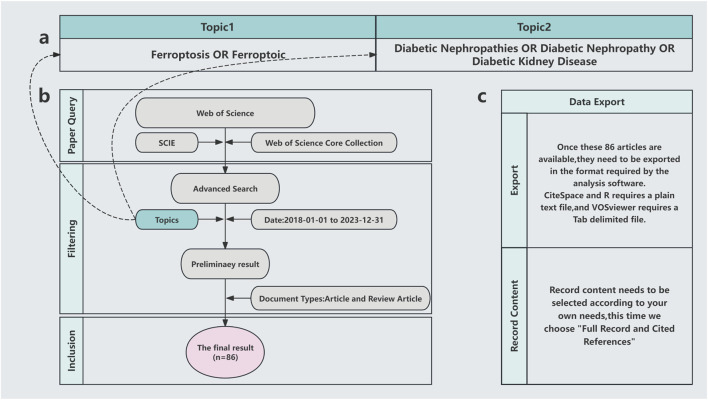
Literature search methods. **(A)** Subject words; **(B)** Search process; **(C)** Export format.

All publications analyzed in this study were sourced from the Science Citation Index Expanded, the core collection database for the Web of Science. We covered the period from 1 January 2018 to 31 December 2023, with 2018 selected as the starting point for literature retrieval because ferroptosis was first identified in 2012, and no studies linking ferroptosis and DN were published before 2018 (based on our search criteria). Only original research articles and review papers were included. The search strategy used the following queries: [TS = (ferroptosis) OR TS = (ferroptotic)] AND TS = (“Diabetic Nephropathies” OR “Diabetic Nephropathy” OR “Diabetic Kidney Disease”). The search yielded 86 publications, which were exported as full-record text format for analyses. The search process is shown (Figure 1).

### 2.2 Bibliometric analysis software

Bibliometric analyses were conducted using CiteSpace 6.3. R1, the R package “bibliometrix,” the Bibliometrics online analysis platform, and VOSviewer 1.6.16. CiteSpace and VOSviewer are widely recognized tools for scientific literature analysis. In both platforms, nodes represent analytical units, encompassing keywords, authors, institutions, and other relevant entities. Co-occurrence and cluster analyses were performed to identify key research trends, collaborative networks, and emerging themes in the field.

## 3 Results

### 3.1 Topic modeling

#### 3.1.1 Publication trend analysis

**FIGURE 2:**
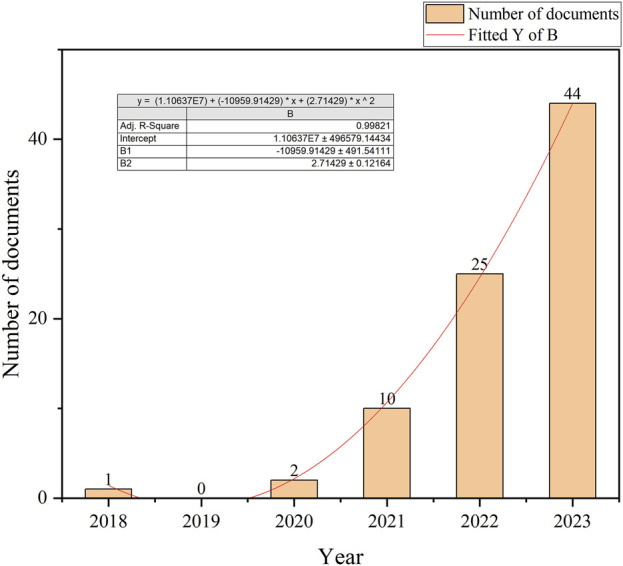
Publication dynamics over the selected search years.

In our search period, publication numbers related to ferroptosis in DN showed a pronounced upward trend, particularly from 2020 to 2023. Notably, between 2018 and 2020, publication numbers remained minimal, with only a few studies contributing to the field. However, from 2020 to 2023, publication numbers surged exponentially, increasing by several orders of magnitude when compared to earlier years (Figure 2). This trend underscored ferroptosis as a critical research area in the broader academic landscape.

#### 3.1.2 The global research landscape

**FIGURE 3:**
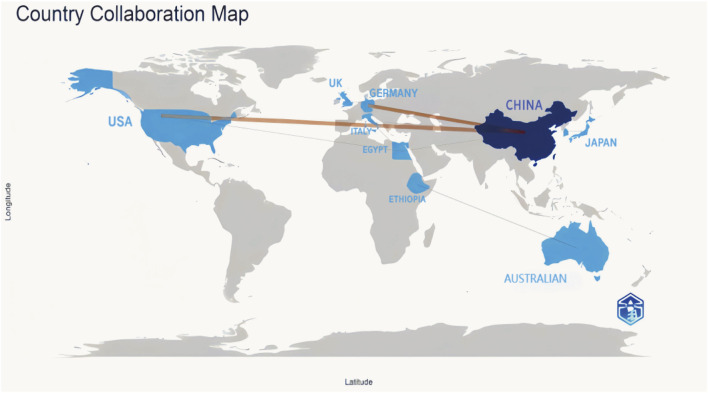
Cooperation Among Countries In the global collaboration map, countries in blue have a significant number of publications, with darker shaded countries having a higher research output. The lines connecting countries show international collaborations, with thicker lines denoting stronger scientific cooperation levels and a higher volume of co-authored publications.

**FIGURE 4:**
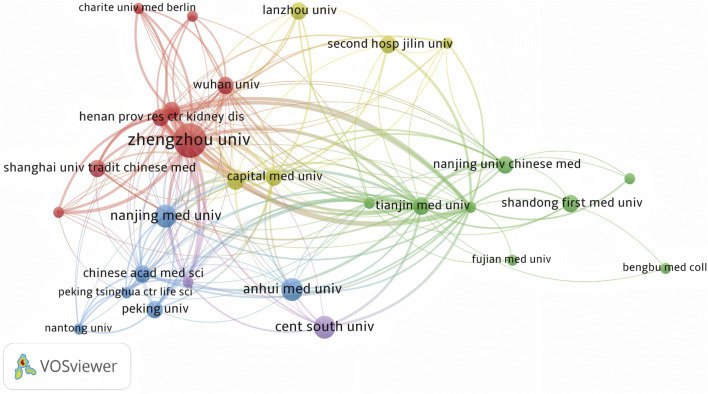
Co-occurrence Analysis of Major Research Institutions Nodes represent research institutions, where larger nodes indicate greater contributions or higher activity levels within the ferroptosis/DN field. Lines connecting nodes show collaborative relationships, and their thickness is proportional to the co-occurrence frequency. Thicker lines denote stronger cooperative ties between institutions, reflecting higher volumes of jointly published research and more extensive scientific collaborations.

**FIGURE 5:**
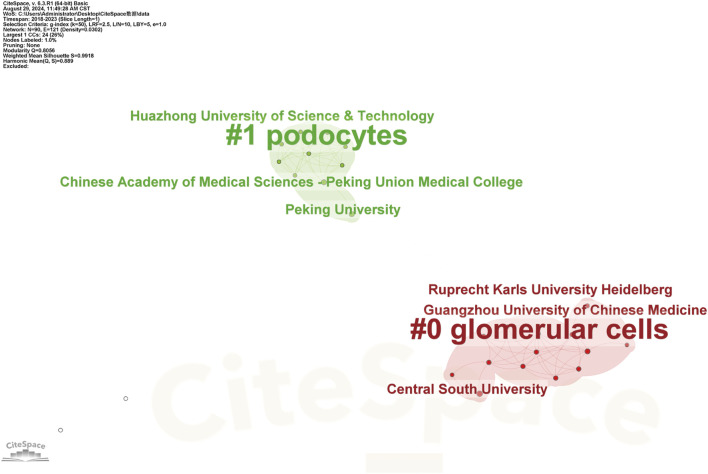
Keyword Clustering Analysis of the Major Research Institutions Research institutions are categorized into clusters, with each defined by common themes or research directions. These clusters may include institutions specializing in biological, chemical, physical, engineering, or information technology research. Each cluster has a corresponding label or representative keyword, clearly indicating the predominant research focus within that group, as demonstrated in CiteSpace’s keyword clustering analysis.

Globally, China emerged as a leading contributor to ferroptosis research in DN, accounting for over 60% of publications from 2018 to 2023. This dominance was exemplified by Zhengzhou University, which contributed five publications in this field. Key international collaborations primarily involved China, the United States of America, and Germany, forming the most substantial global research network (Figure 3). Institutional analysis revealed that Anhui Medical University, Central South University, and Nanjing Medical University had pivotal roles in advancing research on renal ferroptosis mechanisms (Figures 4, 5). Among academic journals, *Frontiers in Endocrinology* (nine articles) and *Biomedicine and Pharmacotherapy* (five articles) were primary platforms for disseminating ferroptosis research, while *Free Radical Biology* and *Medicine and Cell Death and Disease* ranked among the most frequently cited journals (Table 1). Thus, these patterns underscored China’s leadership in both research output and collaborative influence in this rapidly evolving field.

**TABLE 1:** The top 15 research institutions by publication number.

Count	Centrality	Year	Institution	Citation times	H-index
5	0	2023	Zhengzhou University	30	15
4	0	2022	Anhui Medical University	19	55
4	0	2018	Central South University	126	84
4	0	2021	Jilin University	87	64
4	0	2021	Nanjing Medical University	36	71
3	0	2021	Capital Medical University	128	52
3	0	2023	Lanzhou University	6	38
3	0	2023	Mudanjiang Medical University	3	10
3	0	2022	Nanjing University of Chinese Medicine	64	39
3	0	2022	Shanghai University of Traditional Chinese Medicine	38	41
3	0	2021	Tianjin Medical University	182	54
3	0	2022	Wuhan University	61	74
3	0.01	2022	Guangzhou University of Chinese Medicine	47	46
3	0.06	2022	Chinese Academy of Medical Sciences - Peking Union Medical College	26	14
3	0.06	2022	Peking University	21	92

#### 3.1.3 Author and co-cited author analyses

**FIGURE 6:**
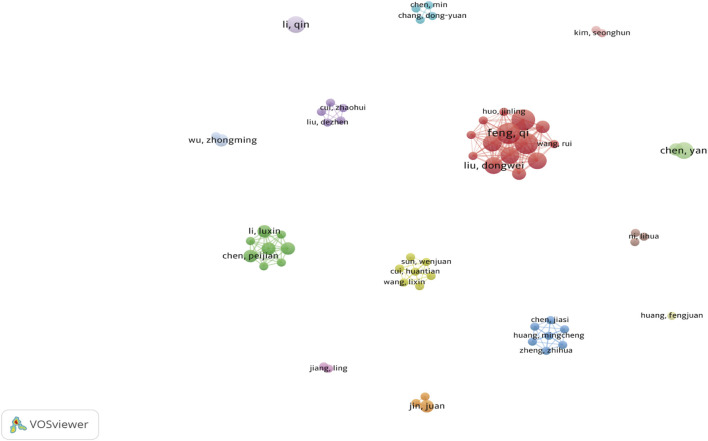
Author Co-occurrence Each node represents an author, while lines show co-occurrence relationships between pairs of authors. Nodes with the same color form a cluster, signifying a specific research direction, collaborative team, or cooperation group.

**FIGURE 7:**
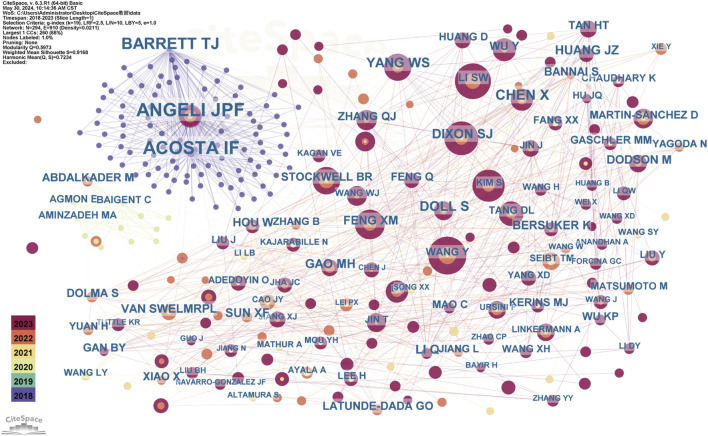
The Co-occurrence of Co-cited Authors Each node represents an author, with the same color nodes indicating frequent co-citations. This suggests a potential alignment between authors’ research topics or thematic focus. The color gradient surrounding each node may reflect temporal variations in citation frequency. In color-based clustering, authors with similar citation patterns are grouped into distinct clusters to form thematic research groups. The category assigned to each cluster indicates that the works cited by this group of authors are closely related, potentially representing a specific research direction or subfield. This clustering method, similar to network analyses applied in academic journal studies within public administration, helps identify influential researchers and emerging trends in ferroptosis/DN research.

Our literature search included 551 authors, with Feng Qi and Chen Yan each contributing four publications. As shown (Figures 6, 7), some authors had close collaborations, whereas others appeared to work more independently. Given the emerging nature of this research field, individual’s publication outputs remained relatively modest. Although variations in publication numbers among authors were minimal, Wang Y, Li SW, Dixon SJ, Kim S, and Feng XM were the five most cited authors (Table 2), underscoring their significant contribution to the field. In terms of scholarly influence, Yang WS and Dixon SJ ranked among the top 10 most cited scholars, demonstrating notable centrality with scores of 0.15 and 0.12, respectively. These values indicated their pivotal roles within the academic community. Notably, José Pedro Friedmann Angeli, despite a relatively modest publication count, had the highest centrality score (0.66). This suggested that this author was the most influential researcher in the specific research block, reinforcing their prominence in the field despite a lower publication volume.

**TABLE 2:** Top 10 authors showing publication numbers and the top 15 authors showing citation numbers.

Items	Rank	Count	Centrality	Year	Cited author/Author
Author	1	4	0	2022	Feng, Qi
	2	4	0	2021	Chen, Yan
	3	3	0	2023	Zhang, Zhen
	4	3	0	2023	Liu, Zhangsuo
	5	3	0	2023	Qiao, Yingjin
	6	3	0	2023	Yang, Yang
	7	3	0	2023	Liu, Jieting
	8	3	0	2023	Pan, Shaokang
	9	3	0	2023	Chen, Peijian
	10	3	0	2023	Liu, Dongwei
Cited author	1	52	0	2021	Wang, Yihui
	2	46	0.01	2021	Li, Shuangwen
	3	40	0.12	2018	Scott J Dixon
	4	37	0	2021	Kim, Seonghun
	5	32	0.03	2021	Feng, Xiaomei
	6	27	0.02	2021	Brent R Stockwell
	7	26	0.15	2018	Wan Seok Yang
	8	22	0.01	2021	Tang, Daolin
	9	19	0.02	2021	Wu, You
	10	18	0	2021	Li, Jialing
	11	18	0.09	2021	Chen, Xin
	12	17	0.66	2018	José Pedro Friedmann Angeli
	13	15	0.05	2022	Zhang, Qijie
	14	14	0.02	2021	Diego Martin-Sanchez
	15	14	0	2022	Wang, Wenjin

#### 3.1.4 Journal and co-cited journal analyses

**FIGURE 8:**
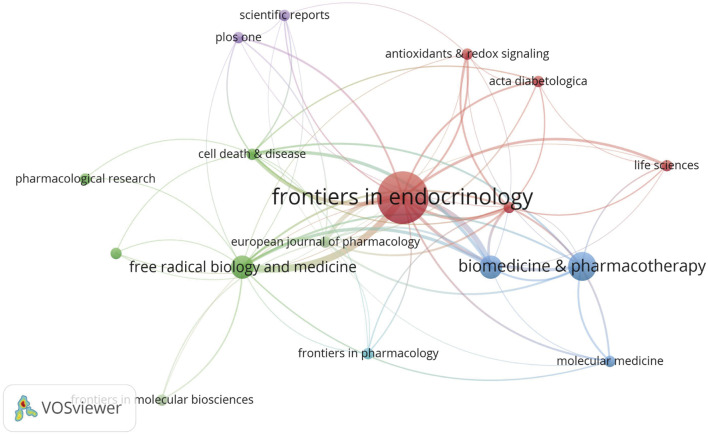
Journal Co-occurrence In network visualization analysis, each node represents an academic journal, and node size corresponds to a journal’s influence and citation frequency. Journals with higher citation rates and a broader academic impact appeared as larger nodes within the network.

In total, 56 journals were included in analyses. As shown (Figure 8), *Frontiers in Endocrinology* had the highest number of publications, with nine articles on ferroptosis and DN (Table 3), followed by *Biomedicine and Pharmacotherapy*. Given that ferroptosis in DN is an emerging research field, the overall publication volume in each journal remained relatively low, with minimal differences between them. This made it challenging to determine which journals were authoritative and representative in the field. However, *Frontiers in Endocrinology* publications were nearly double that of the second-ranked journal, suggesting a possible leading role for this journal in ferroptosis and DN research in the future.

**TABLE 3:** Top 10 journals by volume.

Journals	Rank	Documents
Frontiers in Endocrinology	1	9
Biomedicine and Pharmacotherapy	2	5
Free Radical Biology and Medicine	3	4
Oxidative Medicine and Cellular Longevity	4	4
International Journal of Biological Sciences	5	2
Cell Death and Disease	6	2
European Journal of Pharmacology	7	2
Molecular Medicine	8	2
Life Sciences	9	2
Acta Diabetologica	10	2

**FIGURE 9:**
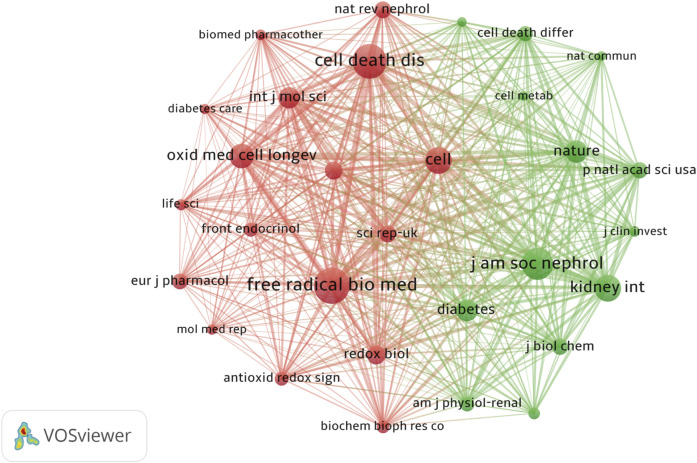
The Co-occurrence of Co-cited Journals Different colors represent distinct clusters, each corresponding to specific research topics or fields. The red cluster was centered around Cell Death and Disease and Free Radical Biology and Medicine, encompassing research directions related to ferroptosis, oxidative stress, and redox signaling. This area is key to understanding the mechanisms through which free radicals contribute to both physiological function and pathological conditions, particularly in the emerging free radical biology field. In contrast, the green cluster centers on Kidney International and the Journal of the American Society of Nephrology, primarily focusing on kidney diseases, with a particular emphasis on DN.

Among the 56 journals, nine were cited over 100 times. Co-citation analysis revealed that *Free Radical Biology and Medicine*, *Cell Death and Disease*, and *Journal of the American Society of Nephrology* ranked among the top three most cited journals. *Cell Death and Disease* demonstrated a steady increase in impact factor and high citation rates, while the Journal of the *American Society of Nephrology* had a significant position in nephrology research. Journal prominence is shown (Figure 9) while the top 10 most cited journals are listed (Table 4).

**TABLE 4:** Top 10 journals in terms of citations.

Cited Journals	Rank	Citations
FREE RADICAL BIOLOGY AND MEDICINE	1	171
CELL DEATH and DISEASE	2	164
Journal of the American Society of Nephrology	3	152
nature	4	106
Kidney International	5	127
CELL	6	127
Oxidative Medicine and Cellular Longevity	7	117
Diabetes	8	104
International Journal of Molecular Sciences	9	102
Redox Biology	10	90

**FIGURE 10:**
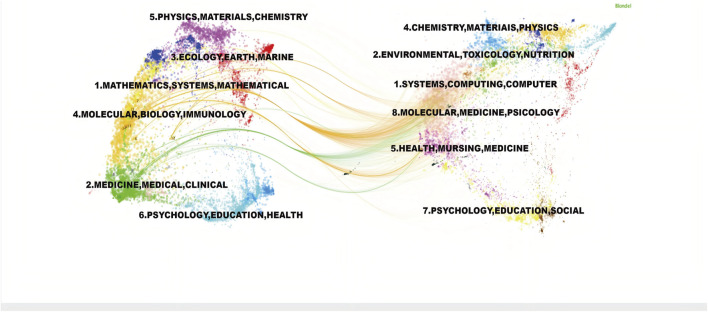
Dual-map Overlay of Journals Cited journals are on the left, citing journals on the right, and the path in the middle represents citation relationships.

To demonstrate the citation relationships between journals, a dual-map overlay was employed to analyze the citation relationships of the journals located on the left and right sides of the map(Figure 10). The green path indicated that journals focusing on molecular studies frequently cited literature from journals in veterinary, animal, and general scientific disciplines. The orange paths showed that journals specializing in molecular biology, environmental studies, toxicology, and nutrition tended to reference journals focusing on biology, immunology, ecology, soil science, and oceanography.

The ferroptosis and DN research field represents a multidisciplinary domain that integrates knowledge from different scientific disciplines. This convergence was particularly evident in the biology impact of ferroptosis-related mechanisms. The field encompassed diverse areas, including biology, medicine, and nutritional science, underscoring the importance of interdisciplinary collaborations in driving continued research advancement in this area.

#### 3.1.5 Emerging trends and cutting-edge research analysis

**FIGURE 11:**
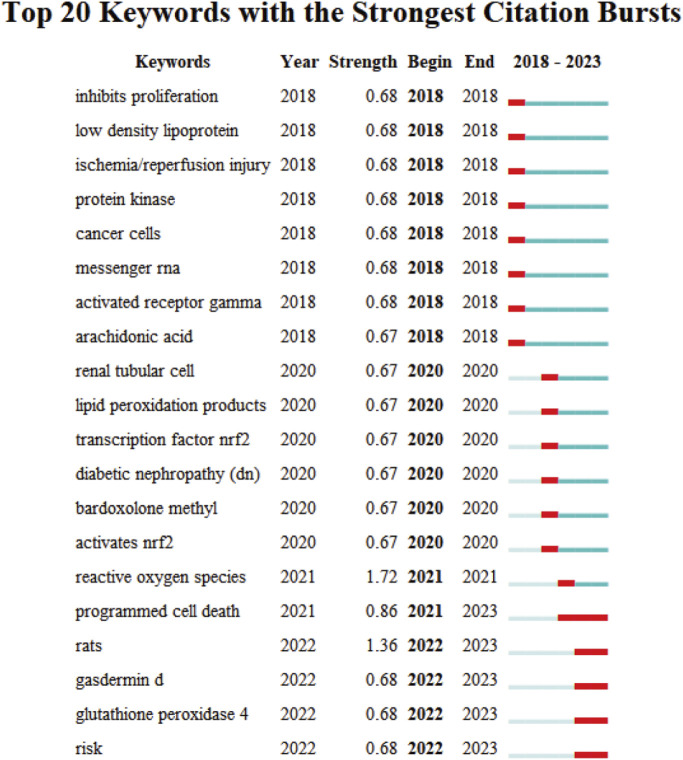
The Top 20 Burst Keywords Red line segments represent a burst keyword, with its position indicating the time period in which it emerged, while its length represents duration.

**FIGURE 12:**
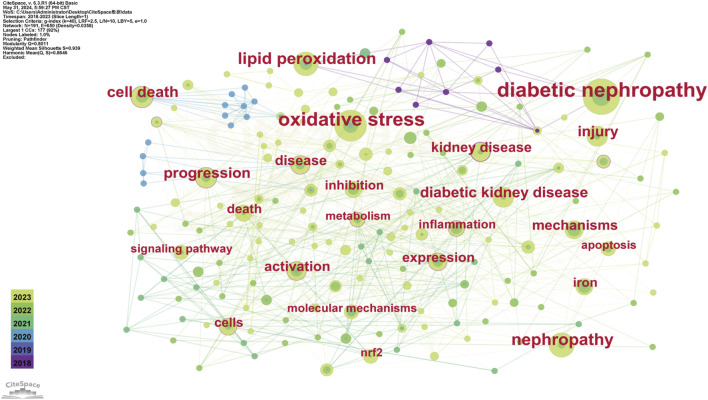
Keyword Co-occurrence Each node represents a keyword and its size reflects keyword frequency occurrence in studies. The color gradient in nodes corresponds to the temporal aspect of keyword emergence. Nodes closer to purple indicate keywords that appear earlier in the timeline (2018), while nodes transitioning toward yellow represent keywords that had gained prominence more recently (2023). The connecting lines show co-occurrence relationships between keywords.

**FIGURE 13:**
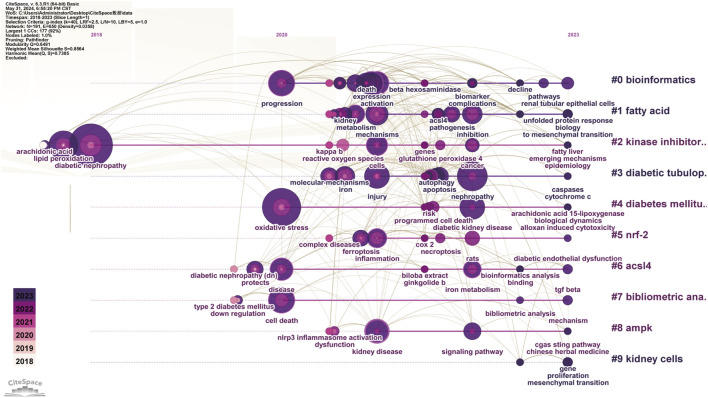
Keyword Timeline Clustering The horizontal axis represents the flow of time, showing the frequency of each keyword at specific time points or years. The vertical axis indicates keyword frequency occurrence in the literature. By analyzing which keywords experienced significant frequency increases, particularly in recent years, emerging research directions and potential future trends were predicted.

**FIGURE 14:**
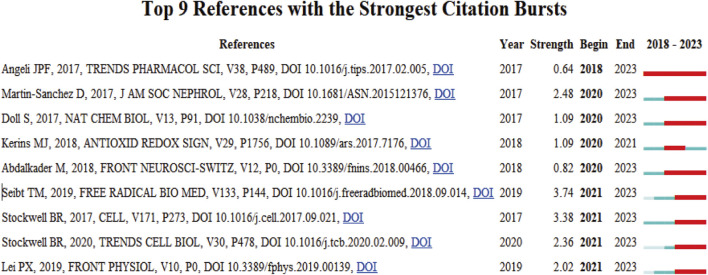
Citation Burst Analysis The red line segment represents a citation burst, and its position corresponds to the period when the citation surge occurred, while red line segment length represents the burst duration.

**FIGURE 15:**
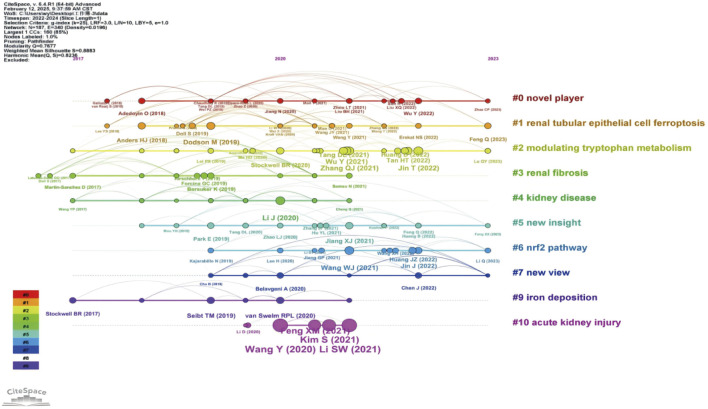
Timeline Clustering of Publications The horizontal axis represents the chronological progression of publications, showing the frequency of each citation at specific points in time or over several years. By analyzing which citations were extensively referenced during a particular period, particularly in recent years, future trends in the field were predicted.

The evolutionary trajectory of ferroptosis in DN research (2018–2023) was represented by multi-dimensional measurement analysis (Figures 11–15). Keyword burst analysis identified “reactive oxygen” as the most intense keyword and “programmed cell death” as the longest-lasting concept (Figure 11), forming a core conceptual framework in the field. Notably, programmed cell death occurred with ferroptosis via p53-mediated solute carrier family 7 member 11 (SLC7A11) regulation and the nuclear factor erythroid 2-related factor 2 (Nrf2)antioxidant pathway. Animal model selection, primarily rats, was based on their robust physiological adaptability, which facilitated mechanistic studies. Key molecular pathways, including the GPX4-mediated regulation of lipid peroxidation and Gasdermin D (GSDMD)-related cross-regulation of pyroptosis, are fundamental pillars in ferroptosis research. The emergence of the “risk” concept in the literature signaled a growing focus on clinical translation. Our co-occurrence network analysis (Figure 12) confirmed that “diabetic nephropathy,” “oxidative stress,” and “lipid peroxidation” formed a stable triangular core (frequency > 50). Also, “renal disease” (centrality 0.27) and “disease progression” were critical bridging concepts, linking mechanistic studies to clinical implications. Time-series clustering (Figures 13–15) revealed a three-stage evolutionary pattern: 1) Foundational Phase (2017–2019): early studies focused on ferroptosis in renal tubular epithelial cells (RTECs) and highlighted NRF2 pathway roles in mitigating oxidative stress. A significant increase in related research projects and funding laid the groundwork for understanding ferroptosis mechanisms. 2) Mechanism Expansion Phase (2020–2023): research expanded to explore tryptophan metabolism and its implications for renal fibrosis, building on foundational ferroptosis knowledge. 3) Interdisciplinary Integration Phase (Post-2021): research paradigms from neurodegenerative diseases were incorporated, suggesting shared ferroptosis-related mechanisms across multiple organ systems. The emergence of clinical transformation concepts, such as “low-threshold intervention strategies,” marked a shift from basic mechanistic analysis to precision medicine approaches.

Each node represents a keyword and its size reflects keyword frequency occurrence in studies. The color gradient in nodes corresponds to the temporal aspect of keyword emergence. Nodes closer to purple indicate keywords that appear earlier in the timeline (2018), while nodes transitioning toward yellow represent keywords that had gained prominence more recently (2023). The connecting lines show co-occurrence relationships between keywords.

### 3.2 Key ferroptosis and cellular iron overload mechanisms

#### 3.2.1 The system Xc^−^/GPX4 axis: the primary antioxidant defense in ferroptosis

**FIGURE 16:**
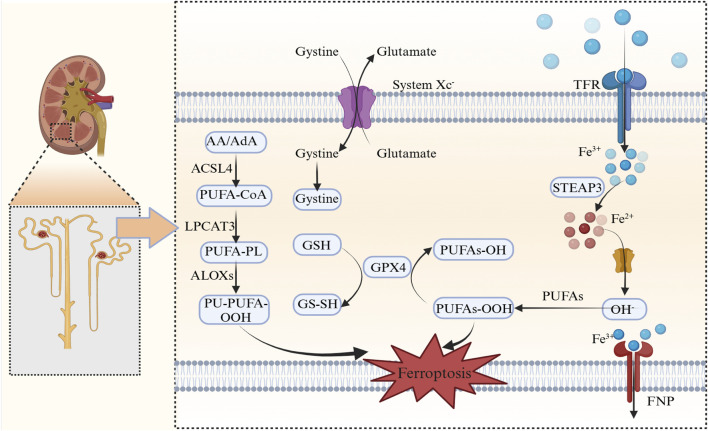
Ferroptosis mechanisms AA/AdA, Arachidonic acid/adrenic acid; ACSL4, Acyl-CoA synthetase long-chain family member 4; PUFA-CoA, Polyunsaturated fatty acid-coenzyme A; LPCAT3, Lysophosphatidylcholine Acyltransferase 3; PUFA-PL, Polyunsaturated fatty acid-phospholipid; ALOXs, Arachidonate lipoxygenases; PL-PUFA-OOH, Phospholipid-polyunsaturated fatty acid hydroperoxide; GSH, Glutathione; GPX4, Glutathione peroxidase 4; PUFAs-OH, Hydroxylated polyunsaturated fatty acids; PUFAs-OOH, Peroxidized polyunsaturated fatty acids; system Xc^−^, Cystine/glutamate antiporter; TFR, Transferrin receptor; STEAP3, Six-transmembrane epithelial antigen of prostate 3; DMT1, Divalent Metal transporter 1; FNP, Ferroptotic Necrotic Pore.

The cystine/glutamate antiporter System Xc^−^ has crucial roles maintaining redox balance, particularly in the central nervous system. This antiporter facilitates cellular cystine uptake, which is a critical step in glutathione (GSH) biosynthesis, and serves as a primary defense against oxidative damage (Figure 16). Pathway regulation occurs in several physiological and pathological conditions, including tumor metabolism and ferroptosis. Glutathione peroxidase 4 (GPX4) is a selenoprotein that uses GSH to reduce cytotoxic phospholipid hydroperoxides to non-toxic alcohols, thereby acting as the primary defense against ferroptosis (Wei et al., 2020; Conrad and Proneth, 2020). Axis dysregulation facilitates lipid peroxide accumulation, leading to ferroptosis initiation. In diabetic retinopathy models, ferrostatin-1 (Fer-1) alleviated hyperglycemia-induced suppression of the GSH/GPX4 axis, thereby restoring redox homeostasis in retinal cells (Shao et al., 2022). *GPX4* knockout or its pharmacological inhibition directly induced ferroptosis across various cancer types (Adamiec-Organisciok et al., 2023). Additionally, in nasopharyngeal carcinoma cells (S18/5-8F), induced ferroptosis by berberine was effectively counteracted by GPX4 overexpression, further underscoring its pivotal role in modulating this iron-dependent cell death pathway (Wu et al., 2024).

#### 3.2.2 The FSP1/CoQ10 system: a parallel antioxidant network

Independent of GPX4, the ferroptosis suppressor protein 1 (FSP1/AIFM2) was shown to use nicotinamide adenine dinucleotide phosphate (NAD(P)H) to regenerate reduced coenzyme Q10 (CoQ10H_2_), which activated radical-trapping to neutralize lipid peroxides (Feng et al., 2020; Gotorbe et al., 2022; Nakamura et al., 2023). This membrane-associated system is in synergy with mitochondrial CoQ10 pools, particularly when GPX4 is dysfunctional (Xavier da Silva et al., 2023). Notably, FSP1 expression was inversely correlated with ferroptosis sensitivity in therapy-resistant cancers, emphasizing its therapeutic relevance as a ferroptosis modulator. Recent studies also indicated that FSP1 inhibitors synergized with GPX4-targeting agents to overcome ferroptosis resistance (Xavier da Silva et al., 2023).

#### 3.2.3 The GCH1/BH4 pathway: phospholipid-specific protection

The GTP cyclohydrolase 1 (GCH1)-tetrahydrobiopterin (BH4) axis has pivotal roles in the selective protection against phospholipid peroxidation, as evidenced by its involvement in antioxidant defense mechanisms and ferroptosis regulation. Under ferroptotic stress, upregulated GCH1 enhanced BH4 biosynthesis, which served as a radical scavenger and stabilized phospholipids containing polyunsaturated fatty acids (PUFAs). This function was essential as BH4 is a crucial cofactor in nitric oxide synthesis, and its disruption potentially elevated ROS production. Studies further reported that BH4 was involved in protein S-nitrosylation and showed increased expression in activated T cells. Dihydrofolate reductase (DHFR) was integral to maintaining BH4 levels, as it recycled dihydrobiopterin to sustain endothelial function, and it also formed a feedback loop that countered lipid peroxidation (Liang et al., 2023; Lee et al., 2021). Previous research has suggested that DHFR deficiency reduced BH4 synthesis and enhanced lipid peroxidation, as observed in aortic valve calcification. Notably, this pathway exhibited tissue-specific activity with pronounced effects in neuronal and renal cells, both of which were particularly vulnerable to PUFA oxidation (Lee et al., 2021).

#### 3.2.4 Iron homeostasis dysregulation: the ferroptosis catalyst

Iron overload amplifies ferroptosis via two primary mechanisms: 1) Metabolic Regulation: Transferrin-bound Fe^3+^ enters cells via transferrin receptor 1 (TfR1)-mediated endocytosis, where it is reduced to Fe^2+^ by six-transmembrane epithelial antigen of prostate 3 (STEAP3) and stored in labile iron pools (LIP). The iron regulatory protein/iron-responsive element-binding protein 2 system was shown to post-transcriptionally regulate ferritin synthesis and iron export via ferroportin (FPN1) (Billesbølle et al., 2020; Keel et al., 2015). 2) Redox Cycling: Iron ions, particularly Fe^2+^, have pivotal roles catalyzing Fenton reactions, where Fe^2+^ reacts with hydrogen peroxide (H_2_O_2_) to generate Fe^3+^, hydroxyl radicals (·OH), and hydroxide ions (OH^−^). These molecules have high reactivity, initiating lipid peroxidation, which is a critical event in many biochemical reactions (Zhang et al., 2023a). Additionally, lipoxygenases were shown to propagate PUFA oxidation in an Fe^2+^-dependent manner, further promoting ferroptosis (Li et al., 2023a).

### 3.3 Ferroptosis effects and mechanisms in renal cells

**FIGURE 17:**
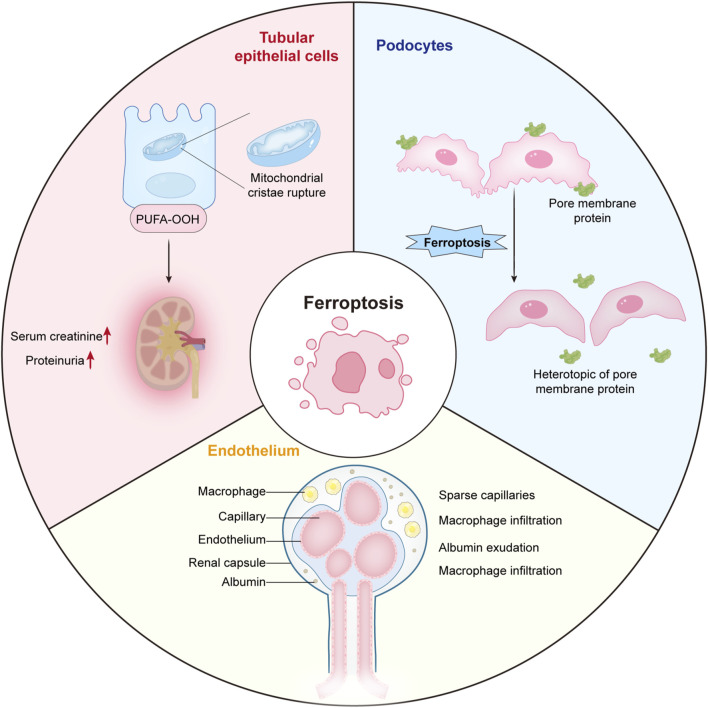
The distribution of ferroptosis in renal tubular epithelial cells, podocytes and endothelial cells.

We demonstrated the distribution of ferroptosis in renal tubular epithelial cells (RTEC), podocytes and endothelial cells (Figure 17).

#### 3.3.1 RTEC injury

Recent studies reported that ferroptosis was a key pathogenic mechanism underlying RTEC injury in DN; the process was driven by iron-dependent oxidative cascades. Research by Liu Zhangsuo’s team at Zhengzhou University highlighted a role for vitamin D receptor activation and its regulatory effects on ferroptosis (Wang et al., 2024a). In the diabetic microenvironment, RTEC degeneration was exacerbated by a self-perpetuating cycle of lipid peroxidation and glutathione depletion, fueled by hyperglycemia-induced oxidative stress, iron overload, and impaired antioxidant defenses (Jin et al., 2023). Mechanistically, chronic hyperglycemia induced mitochondrial dysfunction and activated NADPH oxidase, leading to excessive ROS production (Qiu et al., 2022). The resulting oxidative stress overwhelmed endogenous scavenging mechanisms, intensifying cellular damage. Furthermore, this oxidative milieu synergized with diabetes-associated iron dyshomeostasis, which was characterized by upregulated transferrin receptor expression and suppressed FPN1 levels. These alterations increased LIP levels, creating catalytic conditions for Fenton reactions where redox-active iron (Fe^2+^) reacted with H_2_O_2_ to generate ·OH. These radicals then propagated lipid membrane peroxidation, further exacerbating ferroptotic cell death in renal tubules (Balakrishnan and Kenworthy, 2024).

GPX4 pathognomonic depletion, exacerbated by system Xc^−^ transporter dysfunction under diabetic conditions, critically impaired cells to neutralize phospholipid hydroperoxides. This antioxidant collapse occurred in parallel with the Transforming growth factor-beta (TGF-β)-mediated upregulation of pro-ferroptotic pathways and suppressed Nrf2 signaling, effectively disabling multiple cellular defenses against oxidative injury. Histopathological analysis identified tubular ferroptosis hallmarks, including atrophy and interstitial fibrosis. Ultrastructural studies also identified mitochondrial shrinkage and increased membrane density in affected RTECs–unique morphological features were identified in ferroptosis and were distinguished from other cell death modalities, such as apoptosis or necrosis (Wang et al., 2024a).

The inflammatory microenvironment further amplified ferroptotic cascades via macrophage-derived pro-inflammatory cytokines, which not only enhanced iron retention via heme oxygenase-1 (HO-1) induction, but also depleted cysteine reserves, thereby compromising glutathione synthesis. These molecular disturbances established a vicious metabolic cycle where lipid peroxide accumulation was shown to alter membrane permeability, facilitating excessive iron influx and mitochondrial dysfunction, which in turn generated additional ROS. This self-sustaining RTEC loss mechanism in DN, driven by metabolic abnormalities and oxidative stress, underscored the progressive nature of the disease and highlighted the therapeutic potential of ferroptosis-targeted interventions. Thus, the current evidence has positioned iron chelators and GPX4 activators as promising therapeutic strategies. Preclinical models have also demonstrated that pathway modulation can attenuate both biochemical markers—including ACSL4 upregulation and glutathione depletion—as well as functional parameters, such as serum creatinine levels and proteinuria (Kim et al., 2021; Wang et al., 2022a; Ni et al., 2022; Guo et al., 2023).

#### 3.3.2 Ferroptotic podocyte injury

Iron-dependent cell death in podocytes has emerged as a key driver of glomerular injury in DN. The process was mediated by a cascade of redox imbalances, membrane destabilization, and inflammatory amplification, ultimately resulting in pathological iron accumulation, where excess Fe^2+^ catalyzed Fenton reactions, converting H_2_O_2_ to ·OH. These highly reactive species overwhelmed endogenous antioxidant defenses, leading to an iron-ROS axis that induced catastrophic lipid peroxidation. This peroxidative stress preferentially targeted membrane-embedded PUFAs via non-enzymatic oxidation and acyl-CoA synthetase long-chain family member 4 (ACSL4)-mediated lipid remodeling. Peroxidative phospholipid bilayer degradation also disrupted podocyte structural integrity, resulting in foot process effacement and slit diaphragm protein redistribution (Kruger et al., 2018).

In the diabetic environment, synergistically impaired GSH regeneration occurred via the downregulation of system Xc^−^ and suppressed glutamate-cysteine ligase, which hampered GPX4-mediated lipid hydroperoxide reduction. This antioxidant collapse fostered conditions conducive to ferroptosis, which were further exacerbated by tumor necrosis factor-α(TNF-α)/nuclear factor kappa-light-chain-enhancer of activated B cells (NF-κB)-mediated inflammation, which in turns upregulated iron importers, such as TfR1, while concurrently suppressing FPN1, as shown in osteoarthritis studies (Wang et al., 2023; Jing et al., 2021). Resultant iron retention then established a self-sustaining cycle wherein iron-generated ROS inactivated nephrin and podocin via oxidative modification, compromising the filtration barrier and triggering proteinuria (Alli et al., 2023). Damaged podocytes were shown to further release damage-associated molecular patterns which recruited macrophages. Infiltrating immune cells perpetuated injury by stimulating collagen deposition and α-SMA expression viainterleukin-6 (IL-6)/signal transducer and activator of transcription 3 (STAT3)-mediated pathways, as shown in podocyte injury in focal segmental glomerulosclerosis. Janus kinase 2 (JAK2)/STAT3 signaling was also implicated in this process, thereby establishing links between ferroptosis and progressive glomerulosclerosis (Tao et al., 2018).

Mitochondrial dysfunction was shown to further amplify this cascade by disrupting electron transport chain activity, leading to superoxide leakage that synergized with cytoplasmic ROS pools. Ultrastructural studies identified swollen mitochondria with disrupted cristae in iron-overloaded podocytes, coinciding with upregulated NADPH oxidase 4 (Kahveci et al., 2020). This enzyme is a major ROS source in diabetic glomeruli, with studies confirming its overexpression in podocytes and roles propagating oxidative stress. Clinical correlation analyses also revealed that urinary podocyte-derived extracellular vesicles, enriched in peroxidized lipids, were correlated with disease progression, positioning ferroptosis biomarkers as potential prognostic tools. Emerging therapeutic strategies have targeted this axis, including liproxstatin-1-mediated lipid radical quenching and dexrazoxane-driven iron chelation, which have shown promise in preserving podocyte density and attenuating albuminuria in DN preclinical models (Feng et al., 2023a).

#### 3.3.3 Ferroptosis-driven endothelial dysfunction

Ferroptosis is a central mediator of endothelial injury in DN. This pathology is sustained by a self-reinforcing triad process of oxidative stress, iron dysmetabolism, and inflammatory amplification, as evidenced by a growing body of research on ferroptosis mechanisms in disease progression.

Hyperglycemia-induced mitochondrial uncoupling and subsequent NADPH oxidase activation led to superoxide bursts that overwhelmed endothelial antioxidant defenses. This oxidative stress depleted GSH reserves and impaired GPX4 activity, critical failures that permitted uncontrolled lipid peroxidation. The resulting redox imbalance is further exacerbated by iron overload, a condition frequently associated with diabetes. Within this context, excess Fe^2+^ was shown to catalyze Fenton reactions, converting H_2_O_2_ to ·OH, which preferentially oxidized membrane phospholipids enriched with arachidonic acid and adrenic acid (He et al., 2019). The resultant peroxidative cascade disrupted endothelial barrier function, primarily via caveolin-1 oxidation and vascular endothelial-cadherin internalization. This molecular disruption was clinically manifested as microalbuminuria and capillary leakage, further contributing to vascular complications in DN (Kyselova et al., 2020).

The hypoxic diabetic microenvironment further propagates endothelial ferroptosis through hypoxia-inducible factor 1-α(HIF-1α)-mediated transcriptional reprogramming. Although initially an adaptive response, chronic HIF-1α stabilization paradoxically enhanced HO-1 expression, leading to redox-active iron release from heme catabolism. This liberated iron fueled ROS generation, exacerbating oxidative stress and accelerating ferroptotic cell death. The iron-ROS axis was then shown to act synergistically with tumor necrosis factor-α(TNF-α)/interleukin-1β(IL-1β)-driven inflammation, where activated macrophages secreted S100A8/9 alarmins, which suppressed FPN1 expression, trapping iron in endothelial cells (Guggisberg et al., 2022; Simmen et al., 2019). Simultaneously, advanced glycation end products downregulated system Xc^−^, thereby limiting cystine uptake and glutathione synthesis, effectively disabling the GPX4-mediated repair of oxidized phosphatidylethanolamines. Molecular profiling analysis identified an ACSL4-dominated lipid remodeling program in diabetic endothelial cells, which preferentially incorporated oxidation-prone PUFAs into membrane phospholipids (Singh et al., 2019). This metabolic shift, compounded by a suppressed Nrf2 pathway, established a lipid peroxidation-prone phenotype. Ultrastructural diabetic endothelial ferroptosis hallmarks included fragmented mitochondrial cristae and dilated endoplasmic reticulum, changes that correlated with increased urinary isoprostane levels, which were key peroxidation byproducts linked to declined glomerular filtration rates (Qiao et al., 2023).

Therapeutic strategies targeting this axis demonstrated multimodal efficacy in mitigating endothelial ferroptosis. Deferoxamine, an iron chelator, effectively reduced LIP levels, whereas liproxstatin-1 terminated lipid radical chain reactions, collectively preserving endothelial tight junction integrity in preclinical models. Notably, endothelial-specific GPX4 overexpression attenuated albuminuria and improved peritubular capillary density, further validating ferroptosis as a critical therapeutic target against DN. Emerging biomarkers, such as 4-hydroxynonenal (4-HNE)-modified proteins in circulating endothelial microvesicles, allowed for the non-invasive monitoring of ferroptotic activity. These biomarkers highlighted crucial links between molecular mechanisms and clinical applications, as evidenced by recent studies exploring ferroptosis in cancer treatment and neurodegenerative diseases (Zheng et al., 2021).

### 3.4 Pharmacotherapeutic strategies targeting ferroptosis in DN

Emerging therapeutic interventions mitigating ferroptosis in DN have focused on three core regulatory axes: (1) GPX4 stabilization, (2) Nrf2-mediated antioxidant defense potentiation, and (3) iron homeostasis restoration. Several representative pharmacological agents have shown multimodal efficacy across these pathways, providing promising strategies for managing DN. In particular, established diabetic nephropathy treatments, including angiotensin-converting enzyme inhibitors, angiotensin receptor blockers, insulin therapy, and metabolic disorder medications, have been investigated for their potential roles in modulating ferroptotic processes.

Ginkgolide B (GB) is a terpenoid derived from Ginkgo biloba and exhibits unique anti-ferroptotic activity by regulating proteostasis. Mechanistic studies, including those by Chen et al., reported that GB inhibited ubiquitin-proteasomal GPX4 degradation, thereby preserving its enzymatic function in neutralizing lipid peroxides (Chen et al., 2022). Preserved GPX4 activity is critical for preventing ferroptosis, which is implicated in Parkinson’s disease and cancer progression. In DN models, GB treatment maintained over 60% of physiological GPX4 activity, leading to a 40% reduction in tubular lipid ROS levels, as indicated by decreased 4-HNE + cell levels. Additionally, GB exerted dual suppressive effects on NF-κB and ACSL4 expression, which potentially synergistically enhanced its GPX4-stabilizing properties (Yang et al., 2024a; Yang et al., 2020).

#### 3.4.1 Emodin: an Nrf2 pathway activator

Emodin is a rhubarb-derived anthraquinone and was shown to reverse diabetes-induced Nrf2 suppression by modifying Keap1 cysteine levels, thereby enhancing cellular antioxidant defenses. Transcriptomic profiling indicated that emodin upregulated approximately 78% of Nrf2-regulated genes, including ferritin heavy chain 1 (*FTH1*) and *SLC7A11*, in hyperglycemic podocytes. This transcriptional activation was associated with restored glutathione synthesis and increased iron storage capacity, both of which protected against ferroptotic injury. In addition to its antioxidant properties, emodin also exhibited hypoglycemic effects, reducing fasting blood glucose levels by 30% in Zucker rat diabetes models. Notably, its dual-action mechanisms resulted in a 50% greater reduction in albuminuria when compared to pure Nrf2 activators in comparative studies (Ji et al., 2023).

#### 3.4.2 Dapagliflozin: an iron metabolism modulator

Beyond its role as a sodium-glucose cotransporter 2 inhibitor, dapagliflozin was shown to confer ferroptosis-specific protection by stabilizing SLC40A1, thereby enhancing cellular iron export. In proximal tubule cells, dapagliflozin treatment increased iron efflux by 2.3-fold, leading to a 40% reduction in renal LIP levels. This decrease in intracellular iron levels mitigated Fenton reaction-derived ·OH generation, effectively reducing oxidative stress-mediated tubular injury. Clinical correlation analyses further supported its translational potential, with dapagliflozin-treated patients exhibiting 35% lower urinary 8-isoprostane levels when compared with those receiving standard care (Wang et al., 2024b).

#### 3.4.3 San-Huang-Yi-Shen capsule: a multitarget regulator

The SHYS capsule is a traditional Chinese medicine formulation and a multitarget ferroptosis intervention with system-level effects. Network pharmacology analyses reported 12 bioactive components in SHYS that targeted key ferroptosis regulators, including astragaloside IV (GPX4 stabilization), salvianolic acid B (HO-1 induction), and liquiritin (FPN1 upregulation). In db/db mouse models, SHYS demonstrated comparable reno-protective effects to Fer-1, while uniquely preserving mitochondrial cristae integrity - a structural feature associated with ferroptosis resistance (Lv et al., 2023).

## 4 Discussion

### 4.1 General information

Ferroptosis in DN has emerged as a rapidly evolving research frontier, with an exponential increase in publications since 2020 (Figure 2). Although still in early stages, the area has garnered significant global attention, with China, the United States of America, and Germany leading research efforts and fostering robust international collaborations (Figure 3). Our bibliometric analyses indicated that key journals, such as *Frontiers in Endocrinology* and *Free Radical Biology and Medicine*, were central platforms for disseminating foundational discoveries in this area, highlighting the interdisciplinary nature of the field. Despite these advancements, the mechanistic understanding of ferroptosis in DN remains fragmented, necessitating further investigation into ferroptosis-specific pathways and their interactions with other pathological processes in DN.

As a future research direction, the interplay between ferroptosis and other regulated cell death modalities, particularly pyroptosis, must be elucidated. The simultaneous emergence of keywords, such as “programmed cell death” and “GSDMD” (Figures 11, 12), have underscored the growing interest in cooperative mechanisms underlying renal injury. GSDMD is a defining mediator of pyroptosis (Xiaodong and Xuejun, 2023) and may exacerbate ferroptosis by promoting inflammatory cytokine release, which alters redox homeostasis and amplifies lipid peroxidation. Conversely, ferroptotic cell death may destabilize the cellular microenvironment, thereby priming tissues for pyroptotic activation. Recent studies have suggested that dual-targeting strategies modulating both ferroptosis and pyroptosis regulators could provide novel therapeutic avenues (Quach et al., 2022). Notably, programmed death-ligand 1(PD-L1)/transforming growth factor beta receptor 1 (TGFBR1) dual-targeting small molecule inhibitors and D + T dual-targeting therapy for BRAF-mutated non-small cell lung cancer (NSCLC) generated promising results, supporting the feasibility of combination approaches in modulating disease (Quach et al., 2022; Yang et al., 2024b). Furthermore, integrating metabolomics with spatial transcriptomics could be a potentially powerful approach in elucidating the complex interplay between iron metabolism, oxidative stress, and immune responses in DN progression, as evidenced by the protective effects of traditional medicines such as Huangqi (Astragalus) in DN models.

Current therapeutic strategies targeting ferroptosis in DN have primarily focused on three core axes: (1) enhancing GPX4 stability, (2) activating Nrf2-mediated antioxidant defenses, and (3) restoring iron homeostasis (Table 5). While preclinical models reported promising efficacy, clinical translation research remains limited. While rodent studies have suggested that ferroptosis inhibitors can mitigate DN-related kidney damage, their progression to human clinical trials remains restricted, thereby highlighting challenges in pharmacokinetic optimization and safety profiling. Emerging research has also identified mitochondrial enzymes, such as dihydroorotate dehydrogenase, as critical ferroptosis regulators, suggesting that their targeting could provide novel therapeutic strategies, particularly for cancer treatment (Li et al., 2023b). Additionally, the concept of iron-dependent cell death within the context of anticancer drug-induced injuries has led to the development of ferroptosis inhibitors as potential therapeutic agents. Notably, GPX4-targeting nanobodies were shown to induce ferroptosis in cancer cells, further demonstrating the therapeutic relevance of modulating ferroptosis in different disease contexts. Future research should prioritize large-scale, multi-center clinical studies to validate ferroptosis-targeted interventions, particularly in patients with advanced DN with pronounced iron overload and oxidative damage. Furthermore, cell type-specific vulnerabilities to ferroptosis also warrant further investigation, particularly heightened RTEC sensitivity as a result of their unique metabolic demands.

**TABLE 5:** The effects of different drugs in treating diabetic nephropathy/ferroptosis.

Medicines	Regulators/pathways	Models	Ferroptosis biomarker levels	Citations
Ginkgolide B (GB)	Pa-g/GPX4	Mouse (C57BL/KsJ) Cell (MPC5)	TfR1↑, FTH1↑, GPX4↑	Chen et al. (2022), Yang et al. (2024a), and Yang et al. (2020)
Emodin	Nrf2	SD Rat Cell (HK-2)	GPX4↑, SLC7A11↑, FTH-1↑, TFR1↓, MDA↓, 4-HNE↓	Ji et al. (2023)
Dapagliflozin (DAPA)	HIF1 alpha/Ho-1	Mouse (db/db) Cell (HK-2)	GPX4↑, SLC7A11↑ MDA↓, ROS↓	Wang et al. (2024b)
San-huang-yi-shen capsule (SHYS)	Cys/GSH/GPX4	Mouse (C57BL/6)	GSH↑, GPX4↑, MDA↓, ROS↓, 4-HNE↓	Lv et al. (2023)
Glabridine (Glab)	VEGF/AKt/ERK	SD Rat Cell (NRK-52E)	SOD↑GSH↑GPX4↑ MDA↓TFR1↓	Tan et al. (2022)
Quercetin (QCT)	Nrf2/Ho-1	Mouse (C57BL/KsJ) Cell (HK-2)	GPX4↑, FTH-1↑, SLC7A11↑, GSH↑, MDA↓, 4-HNE↓	Zhang et al. (2023b), Feng et al. (2023b), and Zhou et al. (2024)
Aspirin	COX2	Mouse (DBA/2J) Cell (HK-2)	SLC7A11↑, GPX4↑PTGS2↓, TFR-1↓, MDA↓, 4-HNE↓	Wu et al. (2022)
Schisandrin A	TXNIP/NLRP3	Mouse (C57BL/6) Cell (HRGECs)	MDA↓, GSH↑, Cys↑	Wang et al. (2022b)
Epigallocatechin gallate acid (EG)	Nrf2/Ho-1	SD Rat Cell (HK-2, NRK-52, mTEC)	MDA↓, GSH↓, GPX4↑, ROS↓	Yue et al. (2022)

Mechanistic studies using single-cell RNA sequencing could identify ferroptosis-related gene signatures across glomerular and tubular compartments, thereby facilitating the development of precision therapeutics tailored to specific renal cell populations (Lin et al., 2024). Additionally, the development of non-invasive biomarkers—such as urinary peroxidized lipids or extracellular vesicles indicative of iron overload—could enhance early diagnoses and enable the real-time monitoring of ferroptotic activity in patients with DN.

### 4.2 Study limitations

While we provided a comprehensive review of the latest research advancements in ferroptosis and DN research, acknowledging study limitations is crucial for guiding future research directions. The primary study limitation was data collection and sample representativeness. Our study was based on the published literature, which may have introduced some publication bias. Another critical limitation was an incomplete understanding of the underlying ferroptosis mechanisms in DN. Although associations between ferroptosis and disease progression were identified, a comprehensive understanding of the specific molecular mechanisms was lacking. In particular, the interactions between ferroptosis and other pathophysiological processes at DN onset and progression are critical issues that require investigation.

## 5 Conclusion

This bibliometric and mechanistic study highlighted ferroptosis as a key driver of DN progression, primarily mediated by iron-dependent lipid peroxidation, glutathione depletion, and amplified oxidative stress. Our findings revealed that RTECs, podocytes, and endothelial cells exhibited distinct ferroptosis susceptibilities shaped by cell-specific metabolic and molecular landscapes. These vulnerabilities were characterized by GPX4 inactivation, ACSL4-mediated lipid remodeling, and HIF-1α-driven iron dysregulation, which collectively contributed to DN pathophysiology. Emerging therapeutic strategies focusing on restoring antioxidant defenses, iron chelation, and GPX4 stabilization have demonstrated some preclinical efficacy, while traditional medicine agents such as GB and dapagliflozin have shown translational promise.

But despite rapid technological advancements, several critical research gaps remain. For example, the limited clinical validation of ferroptosis inhibitors, the incomplete understanding of interactions with other cell death pathways, and regional biases in collaborative research networks present ongoing challenges. Future efforts should prioritize large-scale clinical trials, single-cell mechanistic profiling, and interdisciplinary integration to highlight molecular insights for precision therapies. By harmonizing global research efforts and leveraging biomarkers, such as urinary peroxidized lipids, this field has the transformative potential to mitigate DN progression using ferroptosis-targeted interventions.
